# Methods of Sentinel Lymph Node Detection and Management in Urinary Bladder Cancer—A Narrative Review

**DOI:** 10.3390/curroncol29030114

**Published:** 2022-02-23

**Authors:** Ankit Sinha, Alexander West, John Hayes, Jeremy Teoh, Karel Decaestecker, Nikhil Vasdev

**Affiliations:** 1 Hertfordshire and Bedfordshire Urological Cancer Centre, Lister Hospital, Stevenage SG1 4AB, UK; alexander.west2@nhs.net (A.W.); john.hayes13@nhs.net (J.H.); nikhil.vasdev@nhs.net (N.V.); 2 S.H. Ho Urology Centre, Department of Surgery, The Chinese University of Hong Kong, Hong Kong, China; jeremyteoh@surgery.cuhk.edu.hk; 3 Department of Urology, University of Ghent Hospital, 9000 Ghent, Belgium; karel.decaestecker@ugent.be; 4 School of Life and Medical Sciences, University of Hertfordshire, Hertfordshire AL10 9AB, UK

**Keywords:** bladder cancer, sentinel lymph nodes, detection, imaging, PLND

## Abstract

Introduction: Detection of lymph node status in bladder cancer significantly impacts clinical decisions regarding its management. There is a wide range of detection modalities for this task, including lymphoscintigraphy, computed tomography, magnetic resonance imaging, single-photon emission computed tomography, positron emission tomography, and fluoroscopy. We aimed to study the pre- and intraoperative detection modalities of sentinel lymph nodes in urinary bladder cancer. Method: This narrative review was performed by searching the PubMed and EMBASE libraries using the following search terms: (“Transitional cell carcinoma of the bladder” OR “urothelial cancer” OR “urinary bladder cancer” OR “bladder cancer”) AND ((“sentinel lymph node”) OR (“lymphatic mapping”) OR (“lymphoscintigraphy”) OR (“lymphangiography”) OR (“lymph node metastases”)). Studies analysing the effectiveness and outcomes of sentinel lymph node detection in bladder cancer were included, while non-English language, duplicates, and non-article studies were excluded. After analysing the libraries and a further manual search of bibliographies, 31 studies were included in this paper. We followed the RAMESES publication standard for narrative reviews to produce this paper. Results: Of the 31 studies included, 7 studies included multiple detection methods; 5 studies included lymphoscintigraphy; 5 studies included computed tomography and/or single-photon emission computed tomography; 5 studies included fluoroscopy; 4 studies included magnetic resonance imaging; and 5 studies included positron emission tomography. Discussion: Anatomical, radioactive, and functional detection modalities have been studied independently and in combination. The consensus is that preoperative detection with imaging helps guide surgical management and intraoperative detection methods help capture any lymph nodes that may have been missed. Each of these types of detection represent their own set of benefits and drawbacks, but there is currently limited evidence to support any change in overall practice to replace conventional staging.

## 1. Introduction

Bladder cancer is the fourth most common cancer in men and the tenth most common in women. It has the highest lifetime cost per patient of all cancer types due to the long-term survival rate and intensive surveillance that is used [1]. Locally advanced disease is optimally managed with radical cystectomy and urinary diversion, which is associated with the worst health-related quality of life among all cancer patients [2,3].

Muscle invasive bladder cancer (MIBC) has a mortality rate of 50% over 5 years despite optimal management and no histopathologic signs of lymph node metastases [4,5]. This suggests half of patients have disease dissemination that is not detected by current staging techniques [6,7]. As lymph node metastases are associated with negative prognosis, radical cystectomy is routinely combined with lymphadenectomy as routine management of MIBC [5,6,8,9]. The extent of lymph node dissection is a highly debated topic, as it represents a fine balance between minimising disease spread with longer surgeries and complications, such as bleeding and lymphocoele development [8].

Although lymph node biopsy is pertinent in bladder cancer, the current templates of limited, standard, extended, and super extended pelvic lymph node dissection (PLND) could be improved through the use of lymph node mapping. This can aid selective resection of invaded LNs similar to the techniques used in breast and melanoma tumours [8]. An important aspect of LN mapping is detection of sentinel lymph nodes (SLNs) to assess nodal disease status. SLNs are thought to be the first draining lymph nodes from the cancer site and therefore reflect the pathologic status of the remaining lymphatic region. SLN biopsy has been shown to be a useful step in evaluating disease spread in melanoma, penile, and breast cancer [9,10].

Currently, there is an unmet need for more sensitive and anatomically accurate detection of bladder cancer and SLNs. Improved disease localisation through the detection of positive lymph nodes may allow early detection and prevention of disease progression. This paper looks to assess the current literature and practice in SLN identification and biopsy in bladder cancer, as well as identifying potential novel methods.

## 2. Materials and Methods

This narrative review highlights the areas where research exists and where ongoing research can be directed. The aim is to succinctly summarise the field regarding sentinel lymph node identification and biopsy in bladder cancer as well as highlight new and novel research and technology. The RAMESES publication standard for narrative reviews was followed to produce this paper [11].

**Figure 1:**
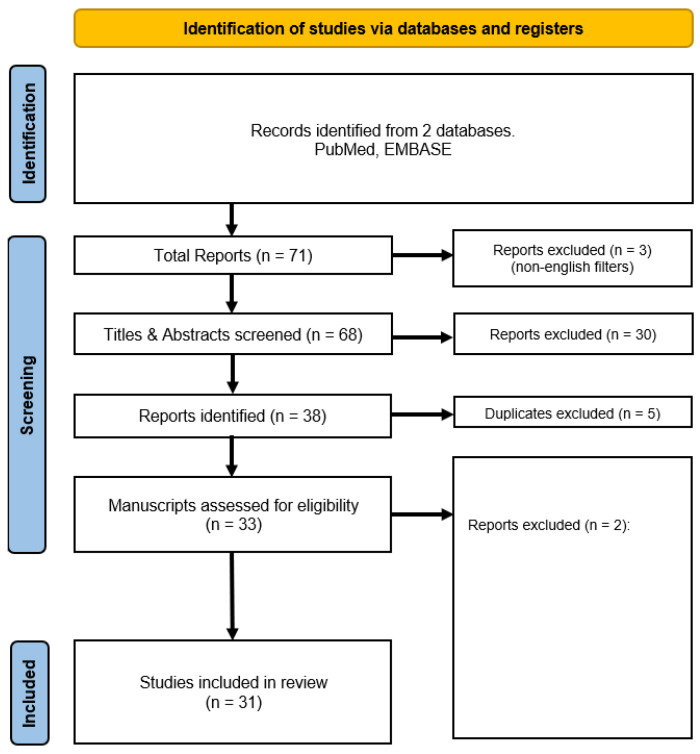
Flow chart of the study selection process. Exclusion criteria for reports included non-English language, books, theses, conference papers, comments, duplicates, and irrelevant content.

PUBMED and EMBASE databases were searched by two independent authors. The search terms used were: “Transitional cell carcinoma of the bladder”, “urothelial cancer”, “urinary bladder cancer”, “bladder cancer”, “sentinel lymph node”, “lymphatic mapping”, “lymphoscintigraphy”, “lymphangiography”, and “lymph node metastases”. No language or date filtering criteria were applied to the initial search, and 53 studies were returned. Manual search of relevant article bibliographies increased this total to 71 studies. The title and abstract were read by authors independently to screen for relevance. Articles that were not agreed upon were then discussed with a third author regarding its inclusion. Exclusion criteria included non-English language, books, theses, comments, conference papers, any duplicates, and papers deemed not relevant after author review. These are summarised in Table 1. As per Figure 1, 31 studies were included in the review after the screening process, summarised in Table 2.

Discussion on techniques, technology, and advances in SN identification and biopsy were extracted. Specific techniques were then grouped for the results section and the relevant papers were presented together. Of the 31 studies that were included in this review, 7 discussed multiple methods of detecting sentinel lymph nodes; 5 discussed lymphoscintigraphy/use of radioactive dye; 5 discussed use of computed tomography (CT) and/or single-photon emission computed tomography (SPECT); 5 studies discussed use of near-infrared fluorescence imaging; 4 studies discussed use of magnetic resonance imaging (MRI); and 5 studies discussed the use of on [^18^F]-fluorodeoxyglucose-positron emission tomography (FDG-PET).

## 3. Results

### 3.1. Lymphoscintigraphy

Lymphoscintigraphy is the detection of the gamma (y) radiation given off by radioactive tracers that are injected into the body. It is performed preoperatively to inform surgical lymph node dissection. The most commonly used radiotracer is ^99m^Technetium, used to label a colloid, such as a sulphur compound or albumin. ^99m^Tc is used as it is less irradiating than some of the early tracers developed (such as ^198^Au) [40]. These were initially detected by planar gamma (y) cameras but this was shown to have a low detection rate of only 23% of patients by Liedberg et al. in their pilot study of 75 patients [18]. Reasons for this could include extensive metastasis obstructing the passage of lymph in the vessels (therefore, preventing the tracer reaching further downstream or forcing it to go to the contralateral side), and radioactive interference from the primary injection, site preventing nearby “hot spots” to be discerned (the “shine-through effect”).

Despite the benefits of preoperative lymphatic visualisation and SLN localisation, there are difficulties with low detection rates. Additionally, the general limitations of radiotracer use (summarised below) prevent planar lymphoscintigraphy replacing current pre- or intraoperative modalities.

Blue dyes (for example, Patent Blue) can also be injected similarly to radiotracers. They add a visually distinct marker to lymph nodes, allowing for easier visual intraoperative detection. They are also cost effective, and application is relatively easy [18]. However, in the study by Liedberg et al., they found there was a low detection rate of 2.67% in their 75-patient cohort [18]. They also found that if it was not correctly applied into the bladder muscular wall, it could stain the entire operating area blue, obscuring resection margins.

Handheld y-probes are another method of detecting metastatic LNs intraoperatively using radiotracers injected similarly to the previous methods. They indicate the areas of highest radioactivity, likely where the radiotracer has accumulated in LNs. Hotspots are displayed on a 2D black background making correlation with the patient’s anatomy challenging. It has been shown to have a higher detection rate than either preoperative lymphoscintigraphy or intraoperative blue dye detection. Indeed, this radio-guided surgery was reported by Liedberg et al. to identify additional SLNs in 7 patients after cystectomy and extended PLND had been performed, showing y-probes’ utility to intraoperatively detect SLNs beyond the PLND template [18]. However, use of handheld y-probes is limited by lack of anatomical information and does not prevent the limitations of metastasis blocking lymph flow and the “shine-through effect”. This makes intraoperative anatomical visualisation necessary [41].

Limitations of radiotracer use apply to all the three methods mentioned in this section. Limitations include radiation exposure to patients and staff, strong legislative control, limited radiotracer availability, expensive detectors and radiotracers, strong initial signals causing “shine through” (a particular concern in bladder cancer as the injection site in the bladder mucosa will be near many of the perivesical LNs of interest), long preparation times, limited half-life of ^99m^Tc (6 h), allergies to blue dyes, viral transmission (through the albumin-based ^99m^Tc colloids as this is derived from human blood), and dependency on nuclear medicine units [42].

### 3.2. Computed Tomography (CT)/Single-Photon Emission Computer Tomography (SPECT)

Lymphadenectomy and histology are widely regarded as the most accurate methods for detecting metastatic deposits in lymph nodes. However, their association with morbidity—and, rarely, mortality—resulted in CT being explored as one of the first alternatives to evaluating and staging bladder cancer [23]. CT and MRI are now routinely utilised to stage advanced disease in candidates for radical cystectomy [43].

The initial use of CT showed low sensitivity and a high false-negative rate as it relied on visibly enlarged lymph nodes to detect disease spread. Of note, there are benign causes of enlarged lymph nodes, and the internal architecture of the nodes could not be discerned. The node itself may also be lost between the “slices” depending on its size [23,31]. Paik et al. reported CT resulted in accurate staging of 54.9% of LNs, with 39% under-staging and 6.1% over-staging [25].

Single-photon emission computer tomography (SPECT) is an imaging modality that uses radiotracers similarly to other methods mentioned above, but images the radiation given off with a gantry of y-cameras, using software to then create a 3D map of radiation hot spots. Studies combining CT with SPECT have the anatomical benefits of CT with the functional benefits of lymphoscintigraphy imaging [44].

The benefits of this technique were demonstrated by Polom et al., who compared these methods in a study of 38 patients with N0 staging according to CT/MRI [22]. The method involved injecting ^99m^Tc-nanocolloid peritumourly via cystoscopy, then Hybrid SPECT/CT was performed 3–8 h later. This technetium tracer is a nanoparticle of radioactive ^99m^Tc, releasing y radiation. Preoperative SPECT/CT has repeatedly been shown to significantly impact surgical approach. Polom et al. found using SPECT/CT altered surgical approach in 7.8% of cases to include resection of lymph nodes outside the PLND template. This method is also effective in melanoma, prostate, cervical, and endometrial cancers [45,46,47,48]. For example, Veenstra et al. using SPECT/CT found additional SLNs in 20% of cases and additional anatomical information in 31% of cases than current methods. This then altered the surgical approach in 29% of cases due to new information [45]. A particular benefit of preoperative SPECT/CT may be in obese patients where intraoperative SLN identification is more challenging, with Lerman et al. reporting an increase in intraoperative identification from 56% to 87% when SPECT/CT was used in these patients [24].

The limitations of SPECT are that it does not solve the issue of false-negative lymph nodes due to metastasis obstruction [24]. It is also a time-consuming and expensive process, requiring earlier admission to allow the SPECT/CT to be performed and interpreted preoperatively, relying on nuclear medicine staff, and has the limitations of radiotracer use, as mentioned earlier.

### 3.3. Positron Emission Tomography (PET)

Positron Emission Tomography (PET) scans use radioactive tracers to create 3D images similar to SPECT, but where SPECT uses tracers that release gamma rays, PET uses tracers that produce positrons which interact with local electrons in the body, releasing energy through photons, which are then detected [44]. The tracer used for this modality is ^18^F-fluorodeoxyglucose (FDG), meaning the scan is often referred to as FDG-PET. This glucose analogue differentiates PET from other imaging modalities, as the FDG acts as a proxy for the metabolism of glucose in the body, meaning PET allows functional imaging rather than structural imaging. Its utility in oncology is widely recognised due to the increased metabolic demand of neoplastic cells causing increased FDG uptake to these areas [49]. This also means that PET can potentially identify metastases in normal sized lymph nodes as it is not reliant on structural changes, unlike conventional CT/MRI. The main use of FDG-PET may be in evaluating lymph nodes deemed suspicious by CT/MRI. This is shown by Dason et al., who studied routine preoperative use of FDG-PET in 185 patients, and found a sensitivity of 92% and a specificity of 91% among 51 suspicious LNs [35]. This combined use of PET and CT/MRI can effectively rule out suspicious LNs. However, they also found low sensitivities (7–23%) in patients found to be N0 on CT. This is likely due to the low burden of disease with smaller median metastasis diameters, meaning less metabolic requirement and FDG uptake. As such, Dason et al. recommended that FDG-PET should not be used routinely in candidates for radical cystectomy, particularly if they lack clinically suspicious lymph nodes [35].

FDG-PET was initially explored to address the weaknesses of CT in detection of sentinel lymph nodes. Large studies by Schöder and Powles have been unable to replicate the encouraging results from smaller studies such as Kosuda et al. and Drieskens et al. [6,40,41,50]. It also has a higher radiation dosage than CT, is less widely available, more expensive, has a longer acquisition time, and has a higher rate of incidental findings, which prompt further invasive testing, cost, and anxiety for patients [35]. FDG is also excreted in the urine which interferes with bladder and perivesical LN assessment. Methods to address this include pre-hydration to dilute urinary tracer, bladder catheterisation to limit tracer accumulation, forced diuresis, and using alternative tracers, such as ^11^C-choline, ^11^C-acetate, and ^11^C-methionine, which demonstrate less urinary excretion. Use of alternative tracers has been shown to be comparable to conventional staging methods [37,43]. However, among 25 patients, Nayak et al. showed a sensitivity of 96% of detecting primary tumours with FDG-PET after forced diuresis with IV Furosemide (20–40 mg) compared with 92% using CT, and a sensitivity of 78% when detecting positive LNs compared with 44% with CT, suggesting that FDG currently remains the best choice [39].

### 3.4. Magnetic Resonance Imaging (MRI)

MRI works by using powerful magnets to detect energy released from proton spin displacement to discern between types of tissue [51]. MRI is another imaging modality which requires changes in lymph node size or aberrant contrast enhancement to detect disease spread. As a result, it generally shows similar results to CT, as both modalities rely on lymph node morphology [31]. Although both CT and MRI are routinely used to stage disease in candidates for radical cystectomy, MRI has been shown to have a higher summary sensitivity—60% (CT—40%) [43]. Methods to address its weaknesses include using diffusion-weighted MRI (DW-MRI) and the use of ultra-small, superparamagnetic particles of iron oxide (USPIO) nanoparticles.

DW-MRI studies the random thermal motion of water molecules in the form of an apparent diffusion coefficient (ADC). The random diffusion of water is generally more restricted in neoplastic tissues than normal tissue due to the higher cell density and the abundance of intra- and extracellular membranes. Therefore, neoplastic tissues have lower ADCs than that of regular tissue. Papalia et al. showed the ADC of metastatic lymph nodes was significantly lower than that of regular lymph nodes [31]. The ADC represents an advantage over CT, and conventional MRI as it is not associated with lesion size. However, DW-MRI does have its own limits. ADC depends on many variables, such as body temperature, tissue pressure, perfusion rate, and magnetic environment, and the interpretation is operator-dependent [31]. Many of these could be addressed by using an in situ comparison from elsewhere in the body, but this requires further study. The results of DW-MRI showed: a sensitivity of 76.4%; a specificity of 89.4%; a positive predictive value (PPV) of 86.6%; and a negative predictive value (NPV) of 71.4% [31]. Despite Papalia et al. discerning a significant difference between metastatic and non-metastatic lymph nodes, the limitations of small sample size and the further study needed to develop an organ-based ADC reference still stand.

Ultra-small superparamagnetic particles of iron oxide (USPIO) can be used with pre- and post-administration MRIs, as well as continuously using handheld magnetometers to guide SLN dissection (similar to how y-probes are used). USPIO are nanoparticles that are injected intravenously which are then carried by the lymph vessels to the nodes. In the nodes, macrophages take them up where the iron oxide’s properties cause a hypointense signal (appears darker). In lymph nodes containing malignant cells, the absence of macrophages results in a relatively hyperintense signal from the lack of iron oxide uptake—which is used in SLN identification [32]. Examples of USPIO include Ferumoxtran-10 (SineremÒ, Guerbet, France) and Sienna+ (Endomagnetics LTD, Cambridge, UK). Sienna+ is being used in breast cancer to localise SLNs with the SentiMag^®^ detection system (Endomagnetics LTD, Cambridge, UK) [42]. Thill et al. found this modality to be equivalent to the current gold standard in breast cancer of using ^99m^Tc radiotracer for detecting SLNs when compared in parallel, intraoperatively, with the same patient population [42]. An added benefit of Sienna+ is that it can be visually identified intraoperatively due to its dark brown colour [42]. To avoid interference with the magnetometer, polymer instruments must be used intraoperatively, preparation time is 20 min compared with up to 29 h required for radiotracers, and the tracer can be injected intraoperatively if using a handheld detection system [42]. This may be applicable to bladder cancer as well.

Many groups studying USPIO-enhanced MRI did not compare their results to that of extended PLND (the gold standard), limiting the accuracy of their negative predictive values. Triantafyllou et al. did compare their results with extended PLND and studied detection of metastases in normal sized lymph nodes (addressing the limitation of spatial resolution in conventional MRI) [33]. They reported a sensitivity of 55.0%, a specificity of 85.5%, a PPV of 57.9%, and an NPV of 83.9% in normal sized lymph nodes (where micro-metastases are more likely to be found). However, studying normal sized lymph nodes may have contributed to the relatively low diagnostic accuracy (77.3%) reported, and having false-negative lymph nodes in 41.5% of patients prevents USPIO-enhanced MRI replacing extended PLND [33]. Deserno et al. similarly showed that USPIO use increased sensitivity from 76% to 96% and NPV from 91% to 98% between pre- and post-contrast MRIs [52].

Birkhäuser et al. combined DW-MRI and use of USPIO in a trial of 75 patients with N0 bladder cancer according to CT/MRI [34]. They demonstrated an improvement to staging for this patient group, with 65–75% of histopathologically proven LN metastases being detected by the 3 readers, with sensitivities from 65% to 75%, specificities from 93% to 96%, and a 25–35% false-negative rate, suggesting improvement over sole DW-MRI use [34]. Comparison of USPIO-DW-MRI with the pre- and post-USPIO enhancement MRI method described by Deserno et al. showed a similar degree of accuracy; however, the USPIO-DW-MRI method required significantly less time to interpret than comparison of the pre- and post-contrast images that the Deserno method required (13 min vs. 80 min per patient, respectively) [32]. Additionally, it may be assumed that the USPIO-DW-MRI images were relatively easy to interpret, as the radiologists who reviewed them were not familiar with the technique.

MRI avoids many of the drawbacks of radiotracer use mentioned above. It also has the benefit of providing results faster than histopathological analysis (interpretation in minutes rather than hours). However, MRI has a high initial cost, slow acquisition, and requires expertise to interpret the results. In addition, USPIO-enhanced MRI, requiring a pre- and post-contrast scan, would further delay progress, as 24–36 h must be kept between the 2 scans.

### 3.5. Indocyanine Green (ICG) Near Infrared (NIR) Fluorescence

Optical imaging using near-infrared (NIR) fluorescence has emerged recently as a safe, real-time method of identifying lymph nodes intraoperatively. It uses intravenous injection of a fluorescent dye indocyanine green (ICG), which has a peak absorption in the NIR wavelength range (820 nm). This means it can be detected by these NIR waves at depths of 5–10 mm in tissues [27]. As the NIR spectrum is also outside visible light, it does not affect the surgical field. ICG’s half-life within the body is around 3–4 min and is excreted by the liver [27].

This technique has already enjoyed some success in identifying SLNs, with Jewell et al. identifying 95% of SLNs in their trial of 227 patients with uterine and cervical cancers by injecting ICG intracervically [53], and Schaafsma et al. achieving a 92% detection rate in patients with high grade bladder cancer when appropriately distending the bladder to maximise lymph drainage [28].

Polom et al. compared NIR ICG fluorescence with ^99m^Tc radiotracer use in detecting SLNs in 47 patients with MIBC [13]. They reported NIR ICG imaging detected all the same lymph nodes as ^99m^Tc, but also managed to detect further LNs that the radiotracer did not in 25.6% of patients, leading to statistically significant differences in outcomes.

NIR ICG fluorescence imaging has the potential to improve bilateral SLN mapping without the drawbacks of a radiotracer, like those of ^99m^Tc, and allows observation of live lymphatic outflow. Its visualisation properties were shown to be more helpful intraoperatively than blue dye by Brouwer et al., who visualised 96.8% of SLNs with ICG in their trial of 65 patients with penile cancer, compared with 55.7% of SLNs using blue dye [41]. Additionally, fluorescence imaging of the dissected SLNs that were not visualised intraoperatively in this study confirmed the presence of fluorescence, suggesting they were obscured by other tissue intraoperatively. They were also studying the utility of combining the optical properties of ICG with the radioactive properties of ^99m^Tc using the combined formulation to use gamma radiation and visual detection to guide sentinel lymph node dissection. This means the current standard of SLN mapping using radiotracer is preserved, with the added benefit of visual fluorescence lasting up to (and possibly beyond) 27 h after tracer injection without additional injection, allowing for a broader range of surgical protocols [41]. ICG may be considered superior to blue dye for the intended purpose of intraoperative visualisation of SLNs.

ICG NIR fluorescence-guided lymph node dissection has limitations as well. It seems to be more effective in patients with lower BMIs, with Jewell et al. finding a significant difference in median BMI of those with successful mapping (30 kg/m^2^) and those with unsuccessful mapping (41.2 kg/m^2^), most likely due to the limited tissue penetration NIR offers [53]. This tissue penetrance is another limitation of ICG NIR fluorescence as it is inferior to that of radioactive signals [41]. Advances in fluorescence imaging may expand the utility of ICG NIR fluorescence in the future.

## 4. Discussion

Detection of sentinel lymph nodes in urinary bladder cancer is an ongoing challenge. Current detection methods using CT and MRI have well documented benefits and limitations. Such anatomical imaging modalities rely on detecting abnormal sizes of lymph nodes. This means that metastases in normal sized lymph nodes are often missed, leading to a proportion of patients being under-staged and contributing to the relatively high false-negative rate (25–40%) of these modalities [6,34,50,54,55]. The false-negative rate is the single most important index of diagnostic performance in sentinel lymph node detection, as positive nodes that are not detected and therefore not dissected may seed further metastases.

Anatomical imaging modalities, such as CT and MRI, rely on discernible, concerning changes that can be seen in lymph nodes, leading to lower sensitivity (particularly in early disease where changes may not be visually obvious). However, their ubiquitous nature and relatively low cost compared with more specialist methods means they remain useful as early steps in the diagnostic pathway.

Functional imaging is possible using FDG-PET and DW-MRI. These modalities are not restricted to detecting visually discernible changes and therefore can be used to detect earlier stages of disease. They are also useful in detecting disease spread where conventional staging has left uncertainty, such as assessing suspicious LNs found on CT/MRI.

Another exciting technique which can augment the results from existing imaging modalities is the use of radiomics studies, which involve extracting quantitative data from medical images and using machine learning to analyse these to answer a clinical question [56,57]. This field has already shown success in differentiating tumour grade in bladder cancer using radiomics features extracted from MRI images [58]. This work has been further expanded to develop and validate MRI- and CT-based radiomics signatures for preoperative prediction of lymph node metastases, demonstrating a favourable discrimination ability in both [59,60]. A similar discrimination ability was also demonstrated in the MRI-reported LN-negative population, which is encouraging, as CT and MRI tend to under-stage patients and may miss LN metastases, as mentioned previously.

Current evidence shows that use of radioactive tracers for lymphoscintigraphy provide most utility when combined with use of handheld y-probes intraoperatively. This is further enhanced when an imaging modality is used to add anatomical detail which is missing from this combination. This could be in the form of preoperative SPECT or combined SPECT-CT, which has been shown to significantly affect management plans in PLND in bladder cancer compared with conventional staging.

Methods that introduce radioactive (for example ^99m^Tc, blue dye), magnetic (for example USPIO), and fluoroscopic (for example ICG) tracers all have the advantage of being able to identify SLNs outside the standard PNLD template, improving dissection yield and modifying the surgical approach to account for these. They can also help address the phenomenon of “skip lesions”, where metastatic LNs are found above the common iliac bifurcation (outside the pelvic region) [61]. This phenomenon may occur due to upstream lymph nodes being obstructed by tumour cells or limitations in the studies, meaning upstream lymph nodes were not detected. Imaging modalities, such as lymphoscintigraphy, SPECT, USPIO-enhanced MRI, and fluoroscopy (where a tracer may flow irrespective of arbitrary anatomical boundaries), would all be beneficial in SLN identification in this scenario.

High false-negative results in many studies may be due to the bilateral nature of bladder lymphatic drainage independent of tumour side (“crossover phenomenon”). This was observed by various studies, and varied between 7% and 45% [4,14,15]. This may lead to concurrent sentinel lymph node appearances along different drainage pathways.

Systematic reviews and meta-analyses in oesophageal, cervical, and breast cancers show that lower-grade tumours are associated with higher detection rates of SLNs, suggesting that SLN biopsy should be limited to lower T stages (T1 and T2) [62,63,64,65], which was also reflected in Zarifmahmoudi et al.’s single-centre experience of 41 bladder cancer patients, with a detection rate of 75% in T3–T4 patients and 94% in T1–T2 patients [20]. However, LN staging in early disease progression cannot be clearly recommended, because, unfortunately, most studies included patients with advanced bladder cancer, meaning further evidence on detection of LN status in earlier disease is limited. Furthermore, many studies did not correlate the clinical lymph node status of the patient with the results from the detection modalities.

Assessment of prognostic factors is essential to guide treatment decisions and patient counselling. Cancer staging is most commonly used as a predictive tool for overall outcomes of bladder cancer, which can feed into the more specific prognostic markers for lymph node dissection, such as lymph node status and number of dissected LNs. An increasing number of dissected LNs demonstrated continued improvement in probability of survival. However, the therapeutic utility of lymph node dissection in bladder cancer and the association of extent of dissection with prognosis have limited and inconsistent evidence [66]. This is further complicated by lymph node density and number of dissected nodes, which are used to define adequate PLND. As Bruins et al. show in their systematic review, performing lymph node dissection confers favourable outcomes compared with no lymph node dissection for patients undergoing radical cystectomy in MIBC, and should be routinely performed for this cohort [67]. Despite weak evidence based on retrospective, non-randomised studies with high risks of bias and confounding, there appears to be some consensus on the extended PLND (e-PLND) showing better recurrence-free survival (RFS) and disease-specific survival (DSS) than lesser degrees of dissection [67,68,69,70]. However, the anatomical boundaries of e-PLND (which some argue is up to the level of the inferior mesenteric artery) are not consistent across studies [71]. Additionally, the first prospective, multicentre phase 3 trial studying lymph node templates failed to show improved outcomes with RFS, DSS or overall survival with e-PLND compared with lesser degrees of dissection [72]. Shariat et al. studied the probability of survival of bladder cancer patients and determined lymph node dissection limited to the true pelvis may be adequate for Ta and Tis stages to provide >90% probability of survival; whereas, an e-PLND may be required for the same effect in patients with T1 or higher cancer [65]. However, it is hoped that an ongoing prospective trial with a larger sample size will be able to clarify the role of e-PLND further and establish clear recommendations regarding PLND extent [73].

Neoadjuvant chemotherapy has been shown to improve overall survival in patients with bladder cancer through phase 3 multicentre trials and meta-analyses [74,75]. However, due to the variable response rate many patients will not benefit from this. This, combined with the difficulty in identifying the cohort who would benefit, means it is not used as often as guidelines may recommend [76]. Therefore, identifying patients who are at high risk of LN metastases through functional and augmented imaging using radiomics or tracers helps identify the patients where e-PLND and neoadjuvant chemotherapy might be appropriate.

The limitations of all the SLN detection studies include the lack of node-to-node correlation between imaging modality and gold standard histopathologic analysis, and differences in study design—the experience of the reporting radiologist, enhancement technique, tracer choice and size, criteria defining positive lymph nodes, administration of neoadjuvant chemotherapy, staging of patient cohorts, non-standardised PLND templates, imaging equipment, and extent of lymphadenectomy [43]. This means that individual discrepancies in detection outcome between modalities cannot be compared, and patients are often used as units of analysis (leading to heterogenous and often incomparable results). This is exemplified by the sensitivity range from 33% to 100% and specificity range from 58% to 100%, found by Crozier et al. in their systematic review and meta-analysis comparing imaging modalities in bladder cancer staging prior to radical cystectomy [43].

A common limitation across most of the studies we included is the issue of being underpowered and mostly monocentric. Additionally, 5 studies that were included in this paper were non-systematic reviews, and 20 out of 31 studies included were heterogenous prospective studies. The variety of methodologies, variability in quality of research, risk of bias, and confounding factors limit the conclusions that can be drawn from them.

There is currently no consensus on routine use of FDG-PET, USPIO-enhanced MRI, or fluoroscopy for nodal staging. While each of these methods have had positive findings with regard to higher sensitivity than CT alone, they are unlikely to replace it currently, due to the speed of results and wide availability CT offers.

Further study is needed to characterise the utility of these anatomical, radioactive, and functional detection modalities (individually and in combination), through independent testing; this is necessary because the current evidence base—largely consisting of non-randomised, heterogenous studies—cannot support changes to surgical or chemotherapeutic strategies. Despite promising initial results from newer modalities using USPIO-enhanced MRI, DW-MRI, SPECT/CT, and ICG NIR fluoroscopy, the current evidence basis limits any clear recommendations to change guidelines for detection of sentinel lymph nodes in bladder cancer. Large, multicentre, prospective trials are needed to confidently support such a change; however, the use of these novel modalities secondary to current methods to determine the status of suspicious lymph nodes has shown success and demonstrated clinical utility.

Recent advancements in technology have seen a rapid development in lymph node mapping methodologies. More work is needed to validate these modalities, but they represent an enticing prospect of more sensitive and anatomically accurate sentinel lymph nodes detection, allowing earlier detection and prevention of disease progression, and—by helping to guide the extent and limits of lymph node dissection during radical cystectomy—improving the quality of this lifesaving procedure.

## Figures and Tables

**Table 1:** Table showing the inclusion and exclusion criteria for data collection.

Inclusion	Exclusion
Articles analysing the effectiveness and outcomes of SLN mapping in bladder cancer	Studies that were not in English language
Any date published	Duplicated articles Books, theses, conference articles, comments
Animal and human studies	Irrelevant content

**Table 2:** This table summarises the studies included in this review.

No.	Author	Year	Study Type	Reference	Imaging Modalities	Number of Patients
1	Zarifmahmoudi et al.	2019	Systematic Review and Meta-Analysis	[12]	Lymphoscintigraphy, CT, SPECT, Fluoroscopy	336
2	Polom et al.	2017	Prospective	[13]	Lymphoscintigraphy, Fluoroscopy	50
3	Lusuardi et al.	2013	Review	[14]	CT, MRI, PET	522
4	Salminen et al.	2016	Review	[15]	CT, MRI, PET	370
5	Liss et al.	2015	Systematic Review and Meta-Analysis	[8]	Lymphoscintigraphy, CT, SPECT, Fluoroscopy	156
6	Nissenkorn et al.	1986	Prospective	[16]	Lymphoscintigraphy, CT	26
7	Aljabery et al.	2015	Retrospective	[17]	PET, CT	54
8	Liedberg et al.	2006	Prospective	[18]	Lymphoscintigraphy	75
9	Marits et al.	2006	Prospective	[19]	Lymphoscintigraphy	14
10	Zarifmahmoudi et al.	2020	Prospective	[20]	Lymphoscintigraphy	41
11	Aljabery et al.	2017	Prospective	[6]	Lymphoscintigraphy	103
12	Sherif et al.	2001	Prospective	[21]	Lymphoscintigraphy	13
13	Połom et al.	2016	Prospective	[22]	CT	38
14	Sherif et al.	2006	Prospective	[4]	CT, SPECT	6
15	Salo et al.	1986	Prospective	[23]	CT	51
16	Lerman et al.	2006	Prospective	[24]	CT, SPECT	157
17	Paik et al.	2000	Retrospective	[25]	CT	82
18	Patel et al.	2016	Review	[26]	Fluoroscopy	N/A
19	Aoun et al.	2018	Systematic Review and Meta-Analysis	[27]	Fluoroscopy	271
20	Schaafsma et al.	2014	Prospective	[28]	Fluoroscopy	21
21	Manny et al.	2014	Prospective	[29]	Fluoroscopy	10
22	Knapp et al.	2007	Prospective	[30]	Fluoroscopy	N/A
23	Papalia et al.	2012	Prospective	[31]	MRI	36
24	Thoeny et al.	2009	Prospective	[32]	MRI	21
25	Triantafyllou et al.	2013	Prospective	[33]	MRI	75
26	Birkhäuser et al.	2013	Prospective	[34]	MRI	75
27	Dason et al.	2020	Retrospective	[35]	CT, PET	185
28	Abrahamsson et al.	2017	Prospective	[36]	PET	88
29	Powles et al.	2007	Review	[37]	CT, PET	N/A
30	Schöder et al.	2004	Review	[38]	PET	N/A
31	Nayak et al.	2013	Prospective	[39]	PET	25
